# Seroprevalence of *Fasciola* sp. and *Toxoplasma gondii* Infections in Rural and Urban Inhabitants of Jolfa County, Northwest Iran

**DOI:** 10.1155/2024/5690707

**Published:** 2024-05-07

**Authors:** Shiva Zeinali, Rasool Jafari, Shahram Khademvatan, Ghorban Sakhaei, Sima Masudi, Shahla Khashaveh, Negar Asadi, Elham Yousefi

**Affiliations:** ^1^Student Research Committee, Urmia University of Medical Sciences, Urmia, Iran; ^2^Department of Parasitology and Mycology, School of Medicine, Urmia University of Medical Sciences, Urmia, Iran; ^3^Cellular and Molecular Research Center, Cellular and Molecular Medicine Research Institute, Urmia University of Medical Sciences, Urmia, Iran; ^4^Department of Epidemiology and Biostatistics, School of Medicine, Urmia University of Medical Sciences, Urmia, Iran

## Abstract

Fascioliasis and toxoplasmosis are the two important zoonotic diseases that are endemic in Iran and share some common transmission routes. The present study is aimed at determining the seroprevalence of human fascioliasis and toxoplasmosis in rural and urban areas of Jolfa County, Northwest Iran. In a cross-sectional study, 600 human sera were collected randomly from humans living in Jolfa County including three cities and 13 villages from 2017 to 2018. Anti-*Toxoplasma* IgG and anti-*Fasciola* sp. IgG tests have been performed using the enzyme-linked immunosorbent assay. Four (0.7%) out of 600 human sera showed positive levels of anti-*Fasciola* IgG. Three out of four seropositive humans were from an urban area, and one (25%) was from rural inhabitants. Considering *T. gondii* infection, 45% of studied human sera were seropositive for anti-*T. gondii* IgG. In conclusion, this is the first study reporting *Fasciola* seropositivity in the area. Based on the findings, human fascioliasis is present in the studied area, Northwest Iran, granted in low prevalence. Considering *T. gondii* seropositivity, the prevalence is high, yet close to the reports from other regions in the province.

## 1. Introduction

*Toxoplasma gondii* and *Fasciola* spp. are completely different parasites; the first one is an obligate apicomplexan intracellular parasite, and the latter is a genus of trematodes or flukes. They are entirely unlike in many aspects; however, both are important zoonotic parasites that are food- and/or waterborne and share some common transmission routes [1]. Toxoplasmosis and fascioliasis have worldwide distribution among animals and humans.

*T. gondii* has a wide spectrum of intermediate hosts and infects almost all vertebrates including humans [2]. *T. gondii* is known to be transmitted by meat-containing tissue cysts (meatborne) and vegetables and fruits (plantborne) contaminated by oocysts [3], consistent contact with soil [4], and cats. Additionally, transmission can also occur congenitally [5] and by organ transplantation [6].

It is estimated that approximately one-third of the human population on the planet is infected by *T. gondii* [2]. Nevertheless, the infection with *T. gondii* is benign in most cases, being asymptomatic or in some patients with signs such as cervical lymphadenopathy or ocular disease, but immunocompromised individuals and fetuses may be severely diseased [2]. Congenital toxoplasmosis can result in devastating consequences for the fetus such as miscarriage, stillbirth, and neonate with ophthalmic and neurological disorders and also long-term effects after birth [5]. In adults, it is believed to be benign, but behavioral changes are thought to occur in chronically infected individuals [7]. In immunocompromised patients such as patients with AIDS, it can be life-threatening causing HIV-related toxoplasmic encephalitis [8]. The routine diagnosis is based on detecting antibodies against *T. gondii*; however, molecular diagnosis is available [9].

Fascioliasis on the other hand is a well-known plant- and waterborne zoonotic parasitic disease of herbivores such as ruminants, equids, and camelids but also omnivore mammals such as swine with economic importance and humans with high medical importance [10]. It is caused by two species, *F. hepatica* and *F. gigantica*, that parasitize the bile ducts in the liver and the gallbladder of ruminants and humans as the definitive hosts. *Fasciola* spp. have a life cycle involving snail (genus *Radix* and *Galba*) as an intermediate host. A resistance phase named metacercaria occurs on the aquatic plants or in the water is a stage that causes human and animal infections commonly by eating freshwater wild or cultivated plants and water containing metacercaria [11].

*F. hepatica* is endemic in Europe, Asia, Africa, the Americas, and Oceania, and *F. gigantica* is found in Africa and Asia [10]. Fascioliasis is an emerging neglected zoonotic infection affecting the health and quality of life in humans. The human infection ranges from asymptomatic to acute or chronic fascioliasis. Nonetheless of the clinical manifestation, the infection can be linked to long-term complications such as liver damage, anemia, and malnutrition [12]. During the prepatent period (early infection), antibody detection by immunological techniques such as ELISA is the only tool available for diagnosis. In the chronic phase, antibody detection, antigen detection, and stool microscopy for detecting eggs are helpful [13].

Iran is one of the endemic countries for human fascioliasis [14] and toxoplasmosis [15], especially in the Caspian Sea basin (North of Iran); both infections are in the highest prevalence among the human population [14, 15]. In 1989 in Gilan Province (north of the country), thousands of individuals had signs of fascioliasis. It was the first largest outbreak of human fascioliasis in the world infecting about 10000 humans. Ten years later, the second outbreak affecting 5000 people occurred in the same region [14]. Considering toxoplasmosis, the seroprevalence is reported as high as 64.7% [16] and 74.6% [17] in the Caspian Sea basin (North of Iran).

It is necessary to have updated information about endemic infectious diseases in every region to be armed for future unwilling events. Because there is no comprehensive study on the seroprevalence of human fascioliasis and toxoplasmosis in Jolfa District, Northwest Iran, the present study is aimed at determining the prevalence of the infection in the area.

## 2. Materials and Methods

The present study was carried out on the serum samples collected for the previously published study on the seroprevalence of cystic echinococcosis in Jolfa County [18], which was reconsidered and approved by the Vice-Chancellor of Research and Technology, Urmia University of Medical Sciences, Urmia, Iran (research project number: 2855, ethical code: IR.UMSU.REC.1400.148).

### 2.1. Study Area and Sampling

**Figure 1:**
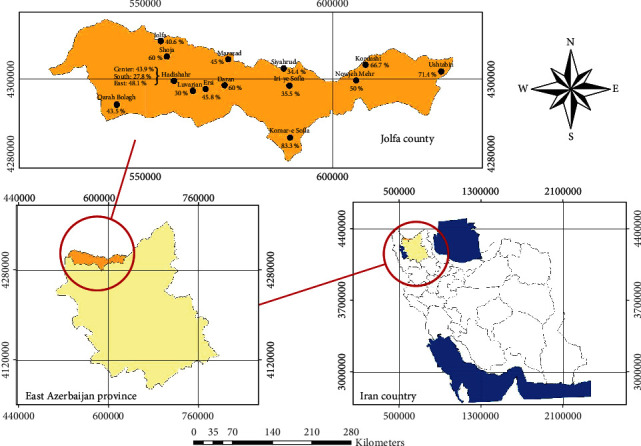
GIS map built for the prevalence of *T. gondii* infection in different studied regions in Jolfa County based on the results of the present study.

Jolfa County is located in the north of East Azerbaijan Province with an area of 1670 km^2^, Northwest Iran. It is located on the south border of Aras River (Figure 1). It is restricted to two countries, Azerbaijan and Armenia. The county's population is estimated at around 61358 humans, 44704 in urban and 16654 individuals in rural districts [18]. The Jolfa County has a semiarid climate.

### 2.2. Sample Collection

This cross-sectional study was conducted with 600 human blood samples collected randomly from humans living in Jolfa County including three cities and 13 villages from 2017 to 2018. The samples were collected from cities including Hadishahr 238 (Central 82, South 79, and East 77), Jolfa 96, and Siyahrud 32 sera and villages including Komar-e Sofla 6, Daran 30, Kordasht 6, Ushtabin 49, Nowjeh Mehr 20, Marazad 20, Qarah Bolagh 23, Luvarjan 10, Ersi 24, Shoja 15, and Iri-ye Sofla 31 samples. Sera were separated from blood and kept frozen at -20°C until the examination.

### 2.3. Determining Anti-*Toxoplasma* and Anti-*Fasciola* IgG

Anti-*Toxoplasma* IgG and Anti-*Fasciola* IgG testing have been performed using the enzyme-linked immunosorbent assay (ELISA kits; Pishtaz Teb, Iran). The tests were carried out based on the manual of the ELISA kits. The sensitivity of 92% and specificity of 93% for anti-*Fasciola* IgG and sensitivity of 100% and specificity of 99% for anti-*Toxoplasma* IgG are claimed by the company. The kits contained positive and negative controls.

### 2.4. Geographic Information System (GIS)

A GIS map of East Azerbaijan Province locating Jolfa County stating sampling areas and *T. gondii* seropositivity was built using ArcGIS v. 10.6 software.

### 2.5. Data Analysis

Data were analyzed by SPSS version 23 (IBM SPSS Statistics for Windows, version 27.0, Armonk, NY: IBM Corp) using respective tests depending on the nature of the data including chi-square, Mann–Whitney, and binary logistic regression tests. *P* value < 0.05 was considered significant.

### 2.6. Limitations of the Study

Analysis of some risk factors related to toxoplasmosis and fascioliasis was a limitation of the present study. These data were not available in the questionnaires, and they could not be retrieved to include in the analysis, such as contact with cats, eating raw meat, and living with livestock.

## 3. Results

### 3.1. Studied Population

Among 600 humans, 352 were female (58.7%) and 248 were male (41.3%). The mean age of the participants was 40.43 years (median = 41, range 2-90 years, STd = 19.02).

### 3.2. Anti-*Fasciola* IgG

Four (0.7%) out of 600 human sera including three females and a male showed positive levels of anti-*Fasciola* IgG. Furthermore, three (0.8%) out of 366 urban (all from southern Hadishahr) and one (0.4%) out of 234 rural (from Daran) inhabitants were seropositive for *Fasciola* spp. Because the number of seropositive humans was low, further data analysis could not be performed. The demographics of positive cases are available in Table 1 as descriptive data.

### 3.3. Anti-*Toxoplasma* IgG

The positive levels of anti-*Toxoplasma* IgG were observed in 270 (45%) human sera. Considering the sex distribution, the seropositivity for *T. gondii* infection was found in 161 (45.7%) and 109 (44%) females and males, respectively. There was a significant relationship between *T. gondii* IgG seropositivity and contact with soil (*P* = 0.002), and living in rural areas (*P* = 0.001) (Table 2). In addition, two out of four (50%) *Fasciola*-infected individuals were coinfected with *T. gondii.*

The lowest *T. gondii* seropositivity was observed in the southern region of Hadishahr, where three out of four cases of fascioliasis were detected, and the highest seroprevalence was observed in Komar village located in the central region of Jolfa County (Table 3 and Figure 1).

The mean age of *T. gondii* seropositive individuals (44.66 years) was significantly higher (*P* < 0.001) compared to seronegative ones (36.96 years).

The IgG concentration was significantly different in different occupations, higher in males and humans that washed raw consumed vegetables with water without using respective disinfectants (Table 4).

## 4. Discussion

In the present study, the seropositivity for anti-*Fasciola* IgG was 0.7%, the majority of whom were from the same area at the moment of sampling, the southern region of Hadishahr. This may reflect an endemic focus on human fascioliasis in the southern part of the city. Considering *T. gondii*, 45% of the studied humans were IgG seropositive. Also, half of *Fasciola*-infected individuals were coinfected with *T. gondii.*

Jolfa County has a semiarid climate suitable for the development of *Fasciola* spp. From different regions of East Azerbaijan Province, there are studies reported *Fasciola hepatica* from livestock [19], but there was no data on the prevalence of *Fasciola* infection in humans in the province, and the present study is the first report of human fascioliasis in Jolfa County, Northwest Iran. The highest human seroprevalence was believed to be reported from a region in Gilan Province (50%). The highest prevalence rate of fascioliasis in animals is reported in the north, and the lowest prevalence is in Central Iran [20].

Heydarian et al. [21] investigated the seroprevalence of human fascioliasis in Lorestan Province, Western Iran, on 1256 humans. They detected anti-*Fasciola* antibodies in 16 individuals (1.3%). Also, no significant differences were reported between infection and age groups, sex, level of education, and occupation; yet significant differences were recorded regarding the location of residency, consuming local freshwater plants, and water sources with seropositivity [21]. In another similar study in the same region, Lorestan Province, Eshrati et al. studied seroepidemiology of human fascioliasis on 1053 humans. They reported *Fasciola* seropositivity in 28 humans (2.66%) among which 18 were females [22]. In the present study, the seropositivity is considerably lower than in the West of Iran. However, in 2016, there was a report from a village in Lorestan Province that resulted in 0.7% of human fascioliasis (6 out of 801) [23], which is exactly similar to the findings of the present study.

There are studies on the seroprevalence of fascioliasis that are reported from different regions of Iran, such as Gorgan, Northeast Iran in 2020, 1.79% (11 out of 612) [24]; Yasuj, Southwest Iran in 2012, 1.8% (18 out of 1000) [25]; Meshkin Shahr (in neighbouring province to ours), Northwest Iran in 2013, 1.96% (9 out of 458) [26]; and Isfahan, Central Iran in 2014, 1.7% (8 out of 471) [27]. Also, human fascioliasis is reported from Mashhad, Northeast Iran [28], and Sistan and Baluchestan Province, Southeast Iran [29]. In Alborz Province, fascioliasis was reported to be significantly lower in patients hospitalized (2.2%) due to COVID-19 compared to healthy controls (4.3%) [30]. In a meta-analysis, the overall prevalence of human fascioliasis in Iran is estimated as 2% (95% CI 1-5) [31].

As it is obvious, human fascioliasis is endemic in most regions of Iran, granted in low prevalence; however, in some areas such as the Caspian Sea basin (North of Iran), there were some outbreaks in the past [14]. Thus, the Jolfa area is not an exception, and based on the results of the present study, human fascioliasis is present in Jolfa County, Northwest Iran, with a close prevalence rate to most of the regions in the country.

Toxoplasmosis is also prevalent in Iran with a wide range of prevalence, higher in warm and humid climates (Caspian Sea basin) and lower in the desert, arid, and colder areas. In a meta-analysis, the overall prevalence of *T. gondii* infection is estimated at 39.3% [15]. In the Jolfa area, only one study could be retrieved by searching databases, which was published in 2005 in the Farsi language by Fallah et al. [32]. They studied anti-*T. gondii* IgG in high school girls in Jolfa using indirect immunofluorescence antibody (IFA) test and reported a prevalence of 21.8%. They also found a significant relationship between having contact with the cat, eating raw liver, and signs (lymphadenopathy, fever, and skin rash) of *T. gondii* infection. In the present study, anti-*Toxoplasma* IgG was observed in 45% of the studied population which is considerably higher than the prevalence reported by Fallah et al. [32]. Furthermore, a significant relationship between *T. gondii* infection and contact with soil and living in rural areas was observed. The lowest *T. gondii* seropositivity was observed in the southern region of Hadishahr, where three out of 4 cases of fascioliasis were also detected, and the highest seroprevalence was observed in Komar Village located in the central region of Jolfa County. The mean age of *T. gondii*-seropositive individuals was significantly higher compared to seronegative ones. In Urmia (Northwest Iran), the seroprevalence of feline toxoplasmosis was reported using a modified agglutination test in 35.3% of stray and household cats [33].

The IgG concentration was significantly different in different occupations (highest in farmers and lowest in students), higher in males and humans that washed raw consumed vegetables with water without using respective disinfectants. This may show a probable relationship between a load of oocysts entering the body with a level of IgG. Contact with cats and eating raw livestock liver were not studied in the present study.

In a study carried out by Rajaii et al. on the seroprevalence of *T. gondii* infection in women of childbearing ages in different regions of East Azerbaijan Province, Northwest Iran, using the IFA test, they studied 1659 women from Tabriz, Maragheh, Ahar, Marand, Sarab, and Mianeh. Of 1659 women, 899 (54.13%) were reported seropositive. They also reported a direct linear relationship between seropositivity and age and higher seropositivity in subjects with lower educational levels and those living in rural regions [34]. In the present study, there was also a similar pattern of some risk factors in Jolfa County, for example, the higher prevalence in rural residents, illiterates, and humans with consistent soil contact and lower prevalence in students, which may show a somehow uniform transmission pattern in East Azerbaijan Province.

## 5. Conclusion

This is the first study reporting *Fasciola* spp. seropositivity in Jolfa County. The southern region of Hadishahr is the area with the highest Fasciola seropositivity, which is concerning regarding the health care system to monitor the area for the infection source to be ready and armed with information on any potential future outbreaks in the area. Considering *T. gondii* seropositivity, the prevalence is high, yet close to the reports from other regions in the province. Furthermore, having contact with soil, living in rural areas, having higher age, and being illiterate increase the risk of being tested seropositive for *T. gondii* in Jolfa County.

## Figures and Tables

**Table 1:** Demographics of the four positive humans for *Fasciola* infection.

Age	Sex	Residential	City/village name	Education	Wild vegetable	Soil contact	Job
71	Female	Rural	Daran	Illiterate	Yes	Yes	Housewife
33	Female	Urban	Hadishahr (South)	Guidance school	Yes	No	Housewife
62	Female	Urban	Hadishahr (South)	Primary school	Yes	Yes	Housewife
46	Male	Urban	Hadishahr (South)	University	Yes	No	Employee

**Table 2:** Odds ratios and *P* values estimated for *T. gondii* seropositivity among different demographic variables in the studied population.

Variable	Anti-*T. gondii* IgG		Total	OR	95% CI	*P*
	Negative	Positive				
Sex						
Female	191 (54.3%)	161 (45.7%)	352	1.075	0.775-1.491	0.665
Male	139 (56%)	109 (44%)	248	1		
Residential						
Urban	221 (60.4%)	145 (39.6%)	366	1		0.001
Rural	109 (46.6%)	125 (53.4%)	234	1.748	1.255-2.435	
Soil contact						
No	223 (59.8%)	150 (40.2%)	373	1		0.003
Yes	107 (47.1%)	120 (52.9%)	227	1.667	1.195-2.326	
Occupation						
Student	73 (80.2%)	18 (19.8%)	91	0.293	0.154-0.557	<0.001
Housewife	121 (48.8%)	127 (51.2%)	248	1.246	0.789-1.970	0.346
Jobless	18 (66.7%)	9 (33.3%)	27	0.594	0.244-1.442	0.250
Farmer	43 (46.7%)	49 (53.3%)	92	1.353	0.772-2.372	0.291
Employee	18 (48.6%)	19 (51.4%)	37	1.253	0.592-2.655	0.555
Self-employed	57 (54.3%)	48 (45.7%)	105	Constant	—	
Washing vegetables						
Water	330 (55.2%)	268 (44.8%)	598	—		0.202
Disinfectant	0 (0%)	2 (100%)	2	—		
Education						
Illiterate	86 (48%)	93 (52%)	179	1.830	1.038-3.225	0.037
Primary school	93 (65%)	50 (35%)	144	0.900	0.497-1.630	0.729
Guidance school	49 (52.1%)	45 (47.9%)	94	1.554	0.826-2.923	0.171
High school	57 (50.4%)	56 (49.6%)	113	1.663	0.904-3.057	0.102
University	44 (62.9%)	26 (37.1%)	70	Constant	—	0.033
Total	329 (54.9%)	270 (45.1%)	600			

**Table 3:** Anti-*T. gondii* seropositivity in different cities and villages of Jolfa County, Northwest Iran.

Residential region	Urban/rural	Anti-*T. gondii* IgG		Total
		Negative	Positive	
Hadishahr (Central)	Urban	46 (56.1%)	36 (43.9%)	82 (100%)
Hadishahr (South)	Urban	57 (72.2%)	22 (27.8%)	79 (100%)
Hadishahr (East)	Urban	40 (51.9%)	37 (48.1%)	77 (100%)
Jolfa	Urban	57 (59.4%)	39 (40.6%)	96 (100%)
Siyahrud	Urban	21 (65.6%)	11 (34.4%)	32 (100%)
Komar-e Sofla	Rural	1 (16.7%)	5 (83.3%)	6 (100%)
Daran	Rural	12 (40.0%)	18 (60%)	30 (100%)
Kordasht	Rural	2 (33.3%)	4 (66.7%)	6 (100%)
Ushtabin	Rural	14 (28.6%)	35 (71.4%)	49 (100%)
Nowjeh Meh	Rural	10 (50%)	10 (50%)	20 (100%)
Marazad	Rural	11 (55%)	9 (45%)	20 (100%)
Qarah Bolagh	Rural	13 (56.5%)	10 (43.5%)	23 (100%)
Luvarjan	Rural	7 (70%)	3 (30%)	10 (100%)
Ersi	Rural	13 (54.2%)	11 (45.8%)	24 (100%)
Shoja	Rural	6 (40%)	9 (60%)	15 (100%)
Iri-ye Sofla	Rural	20 (64.5%)	11 (35.5%)	31 (100%)
Total		330 (55%)	270 (45%)	600 (100%)

**Table 4:** Anti-*T. gondii* IgG concentration in seropositive humans among different demographic variables.

Variable	Mean	Std. deviation	*N*	Mean rank	*P*
Job					0.003
Student	73.1111	69.04209	18	87.86	
Housewife	104.5039	63.16681	127	127.94	
Jobless	78.2222	41.47824	9	98.11	
Farmer	135.6939	68.16439	49	164.86	
Employee	114.4211	58.80695	19	142.61	
Self-employed	119.8542	64.78081	48	147.59	
Sex					
Female	104.8137	64.23426	161	128.41	0.07
Male	119.2018	66.74520	109	145.98	
Residential status					
Urban	110.0759	66.18053	145	134.91	0.894
Rural	111.2560	65.00371	125	136.18	
Education					
Illiterate	117.5054	63.78269	93	143.51	
Primary school	118.0800	64.12865	50	145.12	0.119
Guidance school	90.8444	61.58806	45	111.47	
High school	103.7500	69.11446	56	127.05	
University	120.6923	69.07200	26	148.13	
Vegetable washing with					
Water	111.2463	65.35704	268	136.27	0.06
Disinfectant	27.0000	12.72792	2	32.00	
Soil contact					
No	106.9667	65.70214	150	130.84	0.273
Yes	115.1917	65.27541	120	141.32	

## Data Availability

Data in SPSS format is available and presented by contacting the corresponding author in case of a request.
